# Critical Roles of G3BP1 in Red-Spotted Grouper Nervous Necrosis Virus-Induced Stress Granule Formation and Viral Replication in Orange-Spotted Grouper (*Epinephelus coioides*)

**DOI:** 10.3389/fimmu.2022.931534

**Published:** 2022-07-22

**Authors:** Mengshi Sun, Siting Wu, Shaozhu Kang, Jiaming Liao, Luhao Zhang, Zhuqing Xu, Hong Chen, Linting Xu, Xin Zhang, Qiwei Qin, Jingguang Wei

**Affiliations:** ^1^ College of Marine Sciences, South China Agricultural University, Guangdong Laboratory for Lingnan Modern Agriculture, Guangzhou, China; ^2^ Southern Marine Science and Engineering Guangdong Laboratory (Zhuhai), Zhuhai, China; ^3^ Laboratory for Marine Biology and Biotechnology, Qingdao National Laboratory for Marine Science and Technology, Qingdao, China

**Keywords:** *Epinephelus coioides*, SGs, G3BP1, RGNNV, virus replication

## Abstract

Viral infection causes changes in the internal environment of host cells, and a series of stress responses are generated to respond to these changes and help the cell survive. Stress granule (SG) formation is a type of cellular stress response that inhibits viral replication. However, the relationship between red-spotted grouper nervous necrosis virus (RGNNV) infection and SGs, and the roles of the SG marker protein RAS GTPase-activating protein (SH3 domain)-binding protein 1 (G3BP1) in viral infection remain unclear. In this study, RGNNV infection induced grouper spleen (GS) cells to produce SGs. The SGs particles co-located with the classic SG marker protein eIF3η, and some SGs depolymerized under treatment with the translation inhibitor, cycloheximide (CHX). In addition, when the four kinases of the eukaryotic translation initiation factor 2α (eIF2α)-dependent pathway were inhibited, knockdown of HRI and GCN2 with small interfering RNAs and inhibition of PKR with 2-aminopurine had little effect on the formation of SGs, but the PERK inhibitor significantly inhibited the formation of SGs and decreased the phosphorylation of eIF2α. G3BP1 of *Epinephelus coioides (*named as EcG3BP1) encodes 495 amino acids with a predicted molecular weight of 54.12 kDa and 65.9% homology with humans. Overexpression of EcG3BP1 inhibited the replication of RGNNV *in vitro* by up-regulating the interferon and inflammatory response, whereas knockdown of EcG3BP1 promoted the replication of RGNNV. These results provide a better understanding of the relationship between SGs and viral infection in fish.

## Introduction

In response to different types of external pressure, such as heat shock (1), lack of nutrition, oxidative stress (2), and virus infection (3), eukaryotic cells alter their protein translation. Translation-restricted granular structures form in the cytoplasm to cope with stress conditions and help cells survive, and these complexes are called stress granules (SGs) (4, 5). SGs are also thought to be involved in intracellular signal transduction (6) and selective recruitment of various proteins that regulate different signaling pathways (7). Therefore, SGs play important roles in cell metabolism, cell survival, and viral infection (8).

SGs can inhibit viral replication during the formation process of SGs (9–11). RNA viruses, such as Newcastle disease virus, infect cells and cause them to form SGs, and strong co-localization between viral RNA and SGs has been reported. Retinoic acid-inducible gene I (RIG-I) is also recruited to SGs. The antiviral SG structure induced by Newcastle disease virus is conducive to the recognition of RIG-I and viral RNA, thereby enhancing innate immunity to interferon (IFN) (12). The generation of SGs depends mainly on eukaryotic translation initiation factor 2α (eIF2α)-dependent and eIF2α-independent phosphorylation. SGs must undergo the core process of self-aggregation of RNA binding proteins, such as RAS GTPase-activating protein (SH3 domain)-binding protein 1 (G3BP1) (13), T-cell intracellular antigen 1 (TIA-1) (14), and TIA1-related protein (15). Therefore, the self-aggregation of G3BP1 can be used as a marker of SG formation (16).

G3BP1 belongs to a family of RNA binding proteins that coordinate signal transduction and post-transcriptional regulation of genes in external cells (17). G3BP1 is involved in a variety of cellular processes, such as mediating cell growth, survival (18), proliferation and apoptosis (19). G3BP1 is also closely related to the occurrence and development of tumors (20), G3BP1 was expressed in embryos and in different mature tissues under normal circumstances, and it also was highly expressed in tumor cells (20). G3BP1 contains an N-terminal nuclear transporter two-like domain (NTF2), acidic and proline rich regions, and a C-terminal RNA recognition mode (RRM). The NTF2-like domain of G3BP1 participates in the formation of SGs, mediates the dimerization of G3BP1 itself, and binds to proliferation-promoting proteins (21). Both acidity and proline rich regions are related to protein interaction, and the RRM domain contains two conserved domains, RNP1 and RNP2, which can form special structures and bind to RNA (17).

G3BP1 is also involved in the innate immunity of cells, especially during the process of viral infection. For DNA viruses, G3BP1 can promote the binding of cyclic GMP-AMP synthase (cGAS) and DNA, and the lack of G3BP1 can reduce the binding ability of cGAS and DNA, and inhibit the expression of IFN to a certain extent. These results indicate that G3BP1 plays an important role in the binding of cGAS and DNA (22). For RNA viruses, Yang et al. reported that G3BP1 inhibited the replication of Sendai virus and vesicular stomatitis virus and positively regulated the RIG-I-mediated cellular antiviral response (23). G3BP1 can also interact with various components of SGs. For example, it interacts with Caprin1 to form a complex to promote the formation of SGs, and it interacts with USP10 to inhibit the formation of SGs. Even the G3BP1 mutant that lacks the interaction region can still bind Caprin1 or USP10 to affect the formation of SGs (24).

Grouper (*Epinephelus* spp.) are widely distributed in warm tropical and subtropical waters (25). Because they are rich in high polyunsaturated fatty acids, have great nutritional value, and are suitable for live transportation and temporary feeding, grouper have long been the main fish in China’s fishery consumption market. Red-spotted grouper nervous necrosis virus (RGNNV) infection can cause up to 80% mortality of grouper seedlings, which seriously impacts the long-term development of grouper breeding (26, 27). Therefore, understanding the molecular mechanism of pathogenesis and the interaction between virus and host is crucial for the development of grouper culture. To date, numerous studies have focused on RGNNV biology and its infection mechanism, but little is known about the molecular mechanism of action of the immune system of grouper exposed to the virus. In particular, studies of the relationship between virus infection-induced formation of SGs and SG-related genes and viruses are lacking.

In this study, the molecular mechanism of SG formation in orange-spotted grouper (*Epinephelus coioides*) and the role of related genes during the replication of RGNNV were investigated. The results provide a better understanding of the relationship between SGs and viral infection, as well as a new perspective for developing antiviral strategies to protect cultured fish.

## Materials and Methods

### Cells, Virus and Reagents

Grouper spleen (GS) (28) cell lines were established and maintained in Leibovitz’s L15 medium containing 10% fetal bovine serum (Gibco, Waltham, MA, USA) at 28°C (29). RGNNV was propagated and purified in grouper brain cells (30). The RGNNV titer was determined to be 10^5^ TCID_50_/ml by plaque assay. Virus stocks were maintained at -80°C.

Anti-eIF2α and anti-p-eIF2α were purchased from Cell Signaling Technology (CST). Anti-RGNNV CP polyclonal antibody and Anti-G3BP1 antibody were prepared in our laboratory. Anti-TIA-1 antibody was purchased from Santa Cruz Biotechnology. Anti-β-tubulin (ab6046) antibody was purchased from Abcam company. 2-Aminopurine (hydrochloride) were purchased from GlpBio (United States). GSK2606414 were purchased from GlpBio (United States) and were dissolved in DMSO.

### Cloning of EcG3BP1 and Bioinformatic Analysis

Based on the expressed sequence tag (EST) sequences of EcG3BP1 from the grouper transcriptome, the open reading frame (ORF) of EcG3BP1 was amplified by polymerase chain reaction (PCR) using the primers listed in the **Table 1**. The identity analysis between EcG3BP1 and other G3BP1 sequences was conducted using BLAST searches of the NCBI database (http://www.ncbi.nlm.nih.gov/blast), and the conserved domains or motifs were predicted by the SMART program (http://smart.embl-heidelberg.de/). Multiple alignments of amino acid sequences were conducted using ClustalX 2.1 software. A phylogenetic tree was generated using the neighbor-joining method with 1000 bootstrapping tests within MEGA 6.0 software.

**Table 1:** Primers used in this study.

Primer names	Sequence (5′-3′)
β-actin-RT-F	TACGAGCTGCCTGACGGACA
β-actin-RT-R	GGCTGTGATCTCCTTCTGCA
CP-RT-F	CAACTGACAACGATCACACCTTC
CP-RT-R	CAATCGAACACTCCAGCGACA
RdRp-RT-F	GTGTCCGGAGAGGTTAAGGATG
RdRp-RT-R	CTTGAATTGATCAACGGTGAACA
EcIL-1β-RT-F	AACCTCATCATCGCCACACA
EcIL-1β-RT-R	AGTTGCCTCACAACCGAACAC
EcTNFα-RT-F	GTGTCCTGCTGTTTGCTTGGTA
EcTNFα-RT-R	CAGTGTCCGACTTGATTAGTGCTT
EcIL-8-RT-F	GCCGTCAGTGAAGGGAGTCTAG
EcIL-8-RT-R	ATCGCAGTGGGAGTTTGCA
siRNA1-EcHRI	GCUGGAGCACUUGUGCUUUTT
siRNA2-EcHRI	GCAGACUUCCCGGUAUCUUTT
siRNA3-EcHRI	GCACAACAAAGCCUUCUAATT
siRNA1-EcGCN2	GCAUUUGGUGCUGUAAUUATT
siRNA2-EcGCN2	GCUGGUUACACUGCCCUAUTT
siRNA3-EcGCN2	GGAACUAUGUGAAGGUCAATT
EcISG56-RT-F	CAGGCATGGTGGAGTGGAAC
EcISG56-RT-R	CTCAAGGTAGTGAACAGCGAGGTA
C1-EcG3BP1-F	CGCTCGAGCTATGGTGATGGAGAAGCCAAGTGC
C1-EcG3BP1-R	GCGGATCCCTAGCGCTGGGCAGAGTAGCGGC
EcHRI-RT-F	ATGTTGATGGCTGGTAA
EcHRI-RT-R	CTGGGTTGGTCTCGTA
EcGCN2-RT-F	TGAACTGATAGAAGCCAAGA
EcGCN2-RT-R	GCCTCAAATCCGTAATAAA
siRNA1-EcG3BP1	GCUGGUCGGGCGAGAGUUUTT
siRNA2-EcG3BP1	GCACCGUCGCAAACAAAUUTT
siRNA3-EcG3BP1	GCGGAGGGAAGCUACCAAAT
EcISG15-RT-F	CCTATGACATCAAAGCTGACGAGAC
EcISG15-RT-R	GTGCTGTTGGCAGTGACGTTGTAGT
EcIRF3-RT-F	ATGGTTTAGATGTGGGGGTGTCGGG
EcIRF3-RT-R	GAGGCAGAAGAACAGGGAGCACGGA

### RNA Extraction and cDNA Synthesis

Total RNA from tissues or GS cells were extracted using the SV Total RNA Isolation System (Promega, Madison, WI, USA) according to the manufacturer’s instructions. They were then reversibly transcribed into cDNA using the ReverTra Ace kit (Toyobo, Osaka, Japan).

### qRT-PCR

Quantitative real-time PCR (qRT-PCR) was performed using the SYBR Green I Reaction Mix (Toyobo) in a Roche 480 Real Time Detection System (Roche, Basel, Switzerland). The primers used in the experiment were listed at **Table 1**. Each assay was carried out in triplicate under the following conditions: 1 min for activation at 95°C followed by 40 cycles at 95°C for 15 s, 60°C for 15 s, and 72°C for 45 s. The transcription levels of target genes were normalized to β-actin (reference gene) and calculated using the 2^−ΔΔCT^ method. The data are presented as the mean ± standard deviation.

### Expression Profiles of EcG3BP1 *In Vitro*

To assess the tissue distribution of EcG3BP1 in healthy orange-spotted grouper, the relative expression levels of EcG3BP1 were examined by qRT-PCR. Twelve tissue samples (liver, spleen, stomach, head kidney, kidney, heart, gill, brain, intestine, muscle, fin, and skin) were collected from six groupers, immediately frozen in liquid nitrogen, and stored at -80°C. To explore the response of EcG3BP1 against virus infection, the expression levels of EcG3BP1 were examined in GS cells which were infected with RGNNV. At indicated time points, virus infected cells were collected for RNA extraction and further qRT-PCR analysis.

### Immunofluorescence Assay

GS cells were grown on coverslips (10 mm × 10 mm) in six-well plates. The cells were pretreated with various reagents, such as the translation inhibitor cycloheximide (CHX), the PKR inhibitor 2-aminopurine (2-AP), or the PERK inhibitor GSK2606414) and then the cells were infected with RGNNV. Treated or untreated GS cells were washed with phosphate buffered saline (PBS) and fixed with 4% paraformaldehyde for 1 h at room temperature. After washing with PBS, the cells were permeabilized with 0.2% triton X-100 for 15 min, blocked with 2% bovine serum albumin, and incubated with rabbit anti-G3BP1, mouse anti-CP, mouse anti-TIA-1, or mouse anti-eIF3η serum (1:200) for 2 h at 37°C. The cells were incubated with secondary antibody (Alexa Fluor 555-labeled goat anti-rabbit IgG or Alexa Fluor 488-labeled goat anti-mouse IgG (1:200; Molecular Probe) for 1 h, then cells were stained with 4, 6-diamidino-2-phenylindole (DAPI) for 5 min. The samples were observed under an inverted fluorescence microscope (Zeiss, Oberkochen, Germany).

### Plasmids Construction

In order to investigate the potential function of EcG3BP1 *in vitro*, the subcellular localization of EcG3BP1 was analyzed. The ORF of EcG3BP1 was subcloned into pEGFP-C1 using primers (**Table 1**), and the reconstructed plasmids were confirmed by DNA sequencing.

### Cell Transfection

Cell transfection was performed using transfection reagent (TA) Lipofectamine 2000 (Invitrogen) as described previously (28). In brief, GS cells were grown up to 60-70% confluence in 24-well cell culture plates, and then incubated with the mixture of Lipofectamine 2000 and plasmids according to the manufacturer’s instructions. After 6 h, the fresh normal medium was added and cells were cultured at 28°C for further study.

### Subcellular Localization

To analyze the subcellular localization of EcG3BP1, plasmids including pEGFP-C1 and pEGFP-EcG3BP1 were transiently transfected into GS cells. At 48 h post transfection, cells were fixed with polyformaldehyde, and then stained with 4,6- diamidino-2-phenylindole (DAPI). Finally, samples were imaged under fluorescence microscopy.

### siRNA Knockout of EcG3BP1

To knockdown the expression levels of G3BP1 in GS cells, three siRNAs targeting different sequences of EcG3BP1 mRNA were commercially synthesized by Invitrogen. GS cells were transfected with one of three siRNAs (**Table 1**) or the same volume of the negative control, and then infected with RGNNV or left untreated. At the end of the corresponding incubation period, the total RNA of the extracted cells was detected by qRT-PCR.

### Dual-Luciferase Reporter Assays

GS cells were transiently transfected with the luciferase plasmids (IFN3-Luc or ISRE-Luc) along with the corresponding expression vectors using Lipofectamine 2000 reagent. A total of 50 ng of SV40 was included to normalize the luciferase activities. Cells were harvested to measure the luciferase activities using the Dual-Luciferase^®^ Reporter Assay System kit according to the manufacturer’s instructions.

### Western Blotting

Cells were collected and lysed in radio-immuno-protein assay buffer containing 100 mM NaCl, 0.5% NP-40, 1 mM EDTA, and 20 mM Tris (pH 8.0). Proteins were separated by 10% SDS-PAGE and transferred onto Immobilon-P polyvinylidene difluoride membranes (Millipore, Temecula, CA, USA). The membranes reacted with the indicated primary antibodies: anti-eIF2α, anti-p-eIF2α, anti-RGNNV coat protein (CP) (1:1000 dilution) or anti-β-tubulin (1:1000 dilution). The membranes were washed three times with PBST, and incubated with horseradish peroxidase (HRP)-conjugated anti-rabbit IgG antibodies (1:5000 dilution) for 1 h. Immunoreactive bands were visualized using an enhanced HRP-DAB Substrate Chromogenic Kit (Tiangen).

### Data Analysis

All Statistical analysis were performed by GraphPad Prism 6.0 software. Differences were considered to be statistically significant at *p < 0.05, **p < 0.01.

## Results

### RGNNV Infection Induced SG Formation and Depended on Viral Replication

**Figure 1:**
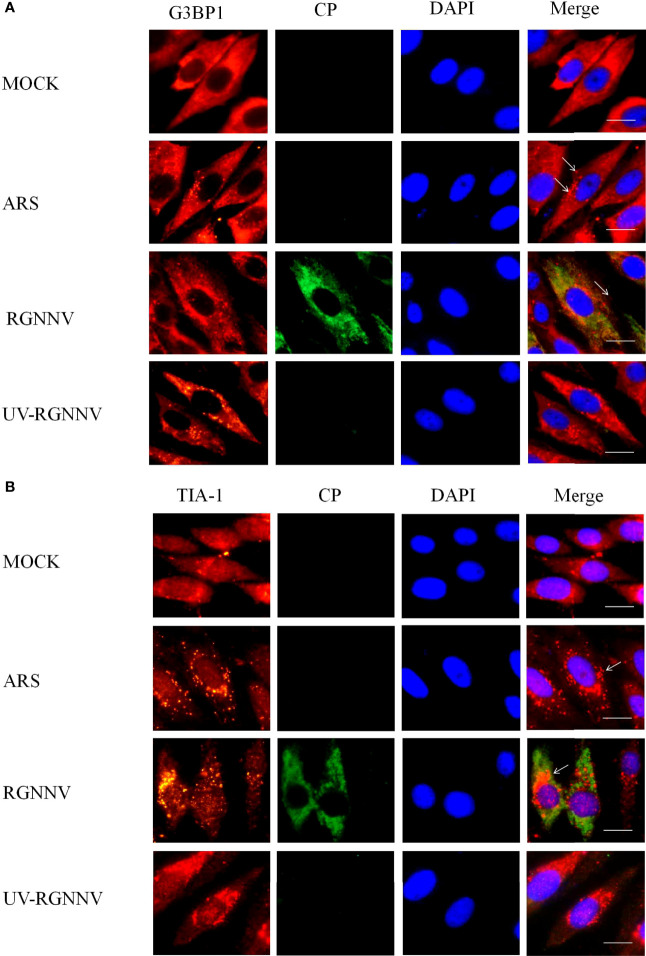
RGNNV infection induced SG production in GS cells. GS cells were fixed at different times post-RGNNV infection (MOI = 2), and the formation of SGs was observed under a fluorescence microscope. G3BP1 (red) indicates the presence of SGs, and CP (green) indicates viral infection. The nuclei were stained with DAPI (blue). Scale bars are 10 µm. **(A)** GS cells were infected with RGNNV or UV-inactivated RGNNV (MOI = 2). The cells were fixed and stained with anti-G3BP1 antibodies and anti-CP antibodies. **(B)** GS cells were infected with RGNNV or UV-inactivated RGNNV (MOI = 2). The cells were fixed and stained with anti-TIA-1 antibodies and anti-CP antibodies.

To investigate whether RGNNV infection induced the formation of SGs in GS cells, indirect immunofluorescence was used to assess the formation of SGs after virus infection. The SG marker proteins G3BP1 and TIA-1 were used to indicate the location of SGs, and the virus was tagged with the antibody to RGNNV CP. To explore whether the formation of SGs depends on the normal replication of the virus, RGNNV was inactivated at 254 nm under aseptic conditions. Four groups were set up in the experiment, and they were negative control group, positive (arsenite treatment, ARS), RGNNV infection group, and UV-RGNNV infection group. The formation of SGs were detected at 24 h after RGNNV infection. As shown in **Figures 1A, B**, both the positive and RGNNV infection groups could induce SGs, whereas the negative control and UV-RGNNV groups could not. These results suggested that RGNNV infection induced SG production in GS cells and the generation of SGs induced by RGNNV infection depended on normal replication of the virus.

### RGNNV Infection Induced the Formation of Classical SGs

**Figure 2:**
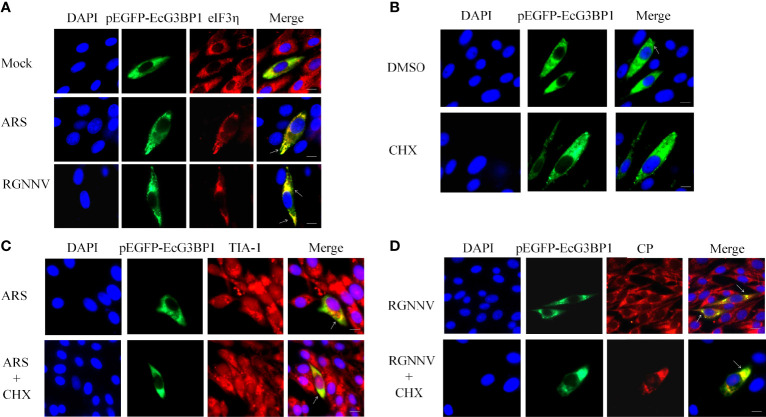
RGNNV infection induced the formation of classic types of SGs. **(A)** GS cells were transfected with pEGFP-EcG3BP1 then treated or not treated with ARS (positive control) for 1 h or RGNNV for 24 h. eIF3η was used to indicate the formation of classical SGs. **(B)** pEGFP-EcG3BP1 was transfected into GS cells. After 24 h, the cells were treated with DMSO or CHX for 1 h. **(C)** GS cells were transfected with pEGFP-EcG3BP1 plasmids. After 24 h, the cells were treated with ARS for 1 h and then CHX for 1.5 h. TIA-1 was used to indicate the formation of SGs. **(D)** pEGFP-EcG3BP1 was transfected into GS cells, which then were incubated with RGNNV at 24 h post-transfection. The cells were treated with CHX for 1.5 h and then analyzed after staining with anti-CP antibodies.

To further investigate the main components and the type of SGs induced by RGNNV infection, G3BP1 was overexpressed in GS cells, and inoculated with RGNNV, and examined after 24 h. Classical SGs were labeled with the eIF3η, and co-localization of eIF3η with EcG3BP1 was detected by indirect immunofluorescence. The ARS treatment group was used as the positive control to detect the formation of typical SGs. In the ARS positive control group and the RGNNV infected group, G3BP1 and eIF3η aggregated to form SGs and were completely co-located, suggesting that RGNNV infection induced the formation of classic SGs (**Figure 2A**). However, the mechanism by which eIF3η was recruited into the SGs is unknown.

It was reported that classic SGs can be depolymerized with CHX (31). To verify the results described above, EcG3BP1 was overexpressed in GS cells, and negative control, ARS positive control, and RGNNV infection groups were set up. After ARS treatment for 1 h or RGNNV infection for 24 h, cells were treated with CHX for 1.5 h. The production of SGs was detected using indirect immunofluorescence, and the co-localization was labeled with TIA-1 or virus RGNNV CP antibody. Overexpression of EcG3BP1 induced cells to form SGs, and it co-located with TIA-1 of the SGs and the CP protein of the virus in all three groups. However, the number of SGs was reduced in CHX-treated cells (**Figures 2B-D**). The result further verified that the formation of classical SGs was induced by RGNNV infection.

### RGNNV Infection Produced SGs Through the PERK Pathway

To determine whether RGNNV induces SG production *via* the eIF2α-dependent classical pathway, GS cells were treated with four eIF2α-related kinase-specific inhibitors or siRNAs, and then detected the formation of SGs using indirect immunofluorescence. The nucleotide analogue 2-AP is an active inhibitor of PKR, and GSK2606414 is the PERK inhibitor. Because HRI and GCN2 have no specific inhibitors, siRNAs of two genes (siHRI and siGCN2) were designed and synthesized, respectively.

**Figure 3:**
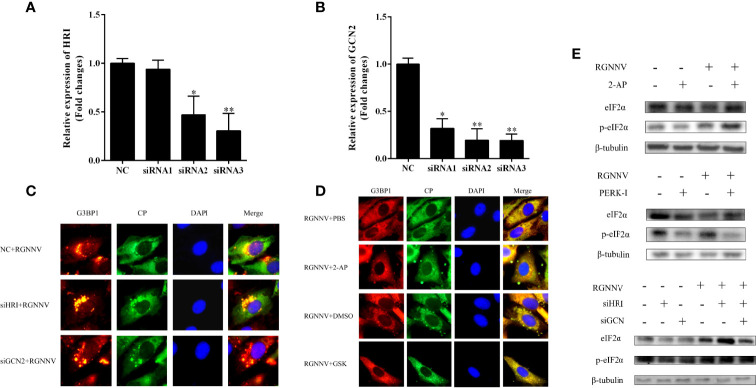
RGNNV infection produced SGs through the PERK pathway. **(A, B)** The knockdown efficiency of siHRI or siGCN2 in GS cells. siHRI and siGCN2 or si-control were transfected into GS cells. After 36 h, the mRNA levels of siHRI and siGCN2 were determined by qRT-PCR. β- tubulin was the internal reference. **(C)** GS cells were transfected with si-control and siHRI or siGCN2 and then infected with RGNNV at 36 h post-transfection. The cells were fixed and stained with anti-G3BP1 antibodies and anti-CP antibodies. **(D)** The cells were treated with DMSO, 2-AP, GSK, or PBS for 1 h, and the medium was changed, and the cells were inoculated with RGNNV for 4 h, and the medium containing DMSO, 2-AP, GSK, or PBS was changed again. **(E)** The phosphorylation level of eIF2α was detected at the protein level. After GS cells were treated with 2-AP or GSK inhibitors or transfected with siHRI and siGCN2, RGNNV-infected cells were phosphorylated. The phosphorylation expression of eIF2α was detected by western blot. *p < 0.05, **p < 0.01.

The interference efficiencies of siHRI and siGCN2 were 70% and 80%, respectively (**Figures 3A, B**). siGCN2 and siHRI were transfected into GS cells, and then the cells were infected with RGNNV. At last, the formation of SGs was detected by indirect immunofluorescence. Compared with the negative control, siHRI and siGCN2 transfection induced the formation of SGs, indicating that the loss of HRI and GCN2 did not affect the RGNNV infection-induced formation of SGs (**Figure 3C**). The cell activity of cells treated with different concentrations of inhibitors were measured. The results showed that the optimal concentration of 2-AP was 0.5 mM and that of PERK-1 was 8.0 mM. PBS and DMSO control groups were set up, respectively. The experimental groups were treated with 2-AP or PERK-1 for 1 h, followed by infection with RGNNV for 4 h, and treated with 2-AP or PERK-1 again for 36 h, and the formation of SGs was detected using indirect immunofluorescence. Both the 2-AP-treated and PBS control groups contained SGs, and the number of SGs did not differ significantly between the two groups. However, compared with the DMSO control, the PERK-1-treated group did not produce SGs. These results indicated that the formation of SGs induced by RGNNV was not related to the effect of the PKR inhibitor, but might be related to the PERK inhibitor (**Figure 3D**). To validate the results, phosphorylation of eIF2α induced by RGNNV under the same conditions was detected by western blot. There was no significant difference in phosphorylation of eIF2α after RGNNV infection in the PRK inhibitor group and in cells transfected siGCN2 and siHRI. However, the phosphorylation of eIF2α was significantly inhibited in the PERK-1 group, which demonstrated that RGNNV induced the formation of SGs through the PERK pathway (**Figure 3E**).

### Sequence Analysis of EcG3BP1

**Figure 4:**
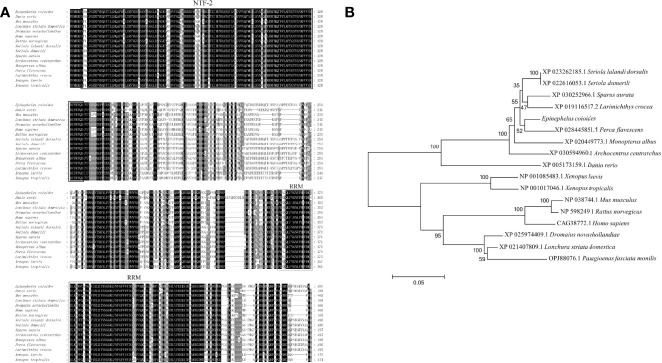
Molecular cloning of grouper EcG3BP1. **(A)** Nucleotide sequence and inferred amino acid sequence of EcG3BP1. Multiple sequence alignments of EcG3BP1 amino acid sequences with other G3BP1 proteins. The conserved domains NTF2 (at positions 7–135) and RRM (at positions 353–432) are boxed with solid lines and dotted lines, respectively. The genetic sequence numbers of these species are as follows: *Danio rerio*, XP_005173159.1; *Mus musculus*, NP_038744.1; *Lonchura striata domestica*, XP_021407809.1; *Dromaius novaehollandiae*, XP_025974409.1; *Homo sapiens*, CAG38772.1; *Rattus norvegicus*, NP_598249.1; *Seriola lalandi dorsalis*, XP_023262185.1; *Seriola dumerili*, XP_022616053.1; *Sparus aurata*, XP_030252966.1; *Archocentrus centrarchus*, XP_030594960.1; *Monopterus albus*, XP_020449773.1; *Perca flavescens*, XP_028445851.1; *Larimichthys crocea*, XP_019116517.2; *Xenopus laevis*, NP_001085483.1; *Xenopus tropicalis*, NP_001017046.1. **(B)** The phylogenetic tree of EcG3BP1 based on amino acid sequences. The data at each node represent the approval rate repeated 1000 times; the scale represents the number of substitutions per kilobase (same below). The GenBank entry number for each species is listed to the right of the species name.

The ORF of EcG3BP1 was amplified from the EST sequence of the spleen transcriptome of *Epinephelus coioides*. Sequence analysis showed that the ORF of EcG3BP1(ON021720) was 1488 bp, encoded 495 amino acids, and the predicted molecular weight was 54.12 kDa. It shared 93.5% and 65.9% identity with gilthead bream (*Sparus aurata*) and human (*Homo sapiens*), respectively. The conserved domains search results showed that EcG3BP1 has a NTF2-like domain and a RRM conserved domain (**Figure 4A**). Phylogenetic tree analysis showed that EcG3BP1 was clustered with the Osteichthyes (**Figure 4B**).

### Tissue Distribution and Subcellular Localization of EcG3BP1

**Figure 5:**
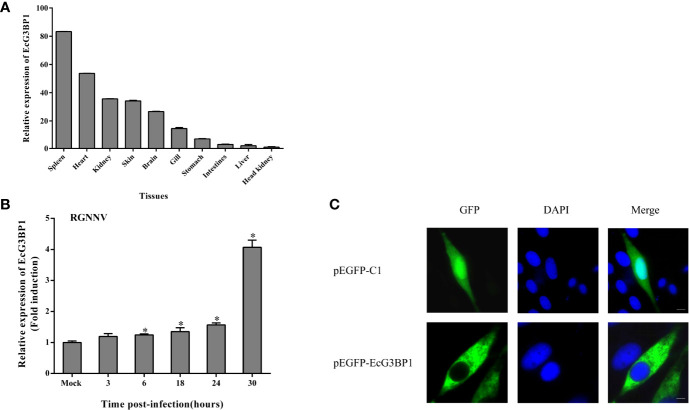
Expression patterns of EcG3BP1. **(A)** Distribution of EcG3BP1 in tissues of healthy grouper. **(B)** Changes of EcG3BP1 expression in GS cells infected by RGNNV. **(C)** The plasmids of pEGFP-C1 and pEGFP-EcG3BP1 were transfected into GS cells and then stained with DAPI. *p < 0.05.

The expression levels of EcG3BP1 in different tissues of healthy grouper were detected by qRT-PCR. EcG3BP1 was distributed and differentially expressed in all examined tissues. EcG3BP1 was mainly expressed in spleen, heart and kidney (**Figure 5A**). To investigate the expression in EcG3BP1 after RGNNV infection, the transcript levels of EcG3BP1 in RGNNV-infected cells were measured using qRT-PCR. The expression of EcG3BP1 gradually increased with the time of RGNNV infection, reaching the highest expression at 30 h, and the expression level was about four times higher than that of uninfected cells (**Figure 5B**).

To investigate the function of EcG3BP1, pEGFP-EcG3BP1 was transfected into GS cells and the localization pattern was observed. Fluorescence microscopy showed that pEGFP-C1 was distributed in both the cytoplasm and nucleus, whereas EcG3BP1 showed cytoplasmic distribution (**Figure 5C**).

### Antiviral Effect of EcG3BP1 on RGNNV Infection *In Vitro*

**Figure 6:**
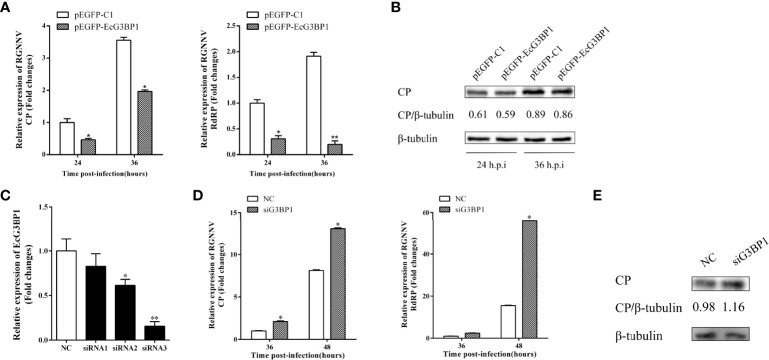
Effect of EcG3BP1 on replication of RGNNV virus. **(A)** EcG3BP1 overexpressing cells were infected with RGNNV. GS cells were collected at 24 and 36 h post-infection, and the relative expression of RGNNV viral genes was examined using qRT-PCR. **(B)** EcG3BP1 was overexpressed in GS cells and infected with RGNNV 24h later. GS cells were collected and the expression level of CP protein was detected by western blot. **(C)** The NC siRNA or three siRNAs targeting EcG3BP1 were transfected into GS cells. The transcription levels of EcG3BP1 were determined at 48 h post-transfection by qRT-PCR. **(D)** GS cells were transfected with NC or siRNAs targeting EcG3BP1. At 48 h post-transfection, GS cells were infected with RGNNV and then collected to measure expression of CP and RdRp of RGNNV. **(E)** The CP protein of RGNNV was detected in transfected cells by western blot. β-tubulin was used as the internal reference. *p < 0.05, **p < 0.01.

To clarify the role of EcG3BP1 in RGNNV replication, pEGFP-EcG3BP1 was transfected into GS cells, and then the cells were infected with RGNNV for 24 h and 36h. The transcriptional levels of RGNNV genes (CP and RdRp) were significantly repressed when EcG3BP1 was overexpressed (**Figure 6A**). When the same treatment was used to detect the protein levels of RGNNV CP at 24 h and 36 h, EcG3BP1 inhibited the protein levels of CP (**Figure 6B**).

Three siRNAs targeting G3BP1 were used to verify the influence of EcG3BP1 knockdown on RGNNV infection. Compared with the NC siRNA, siRNA3 significantly reduced the expression of EcG3BP1 after 48 h, with knockdown efficiency of 84.2% (**Figure 6C**). Next, si-EcG3BP1 and NC siRNA were transfected into GS cells, and the cells were infected with RGNNV after 48 h. At 36 h and 48 h after virus infection, qRT-PCR indicated that knockdown of EcG3BP1 up-regulated the transcription level of RGNNV CP and RdRp (**Figure 6D**). At the same time, GS cells were collected to examine the expression of viral genes by western blotting. Compared to cells transfected with NC siRNA, knockdown of EcG3BP1 promoted RGNNV replication (**Figure 6E**). Together, EcG3BP1 was speculated to inhibit RGNNV replication in GS cells.

### Overexpression of EcG3BP1 Differently Regulated Interferon Immune Response and Pro-Inflammatory Cytokines

**Figure 7:**
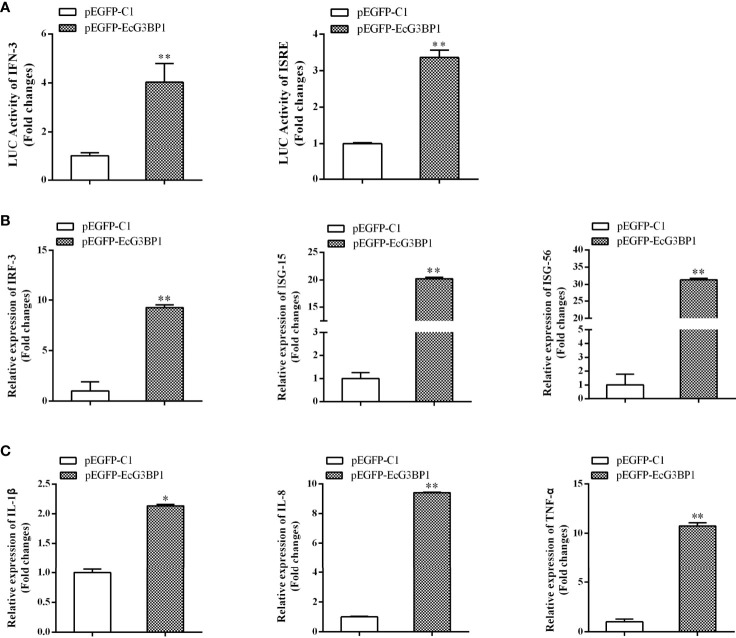
Overexpression of G3BP1 up-regulated interferon immune response and pro-inflammatory cytokines. **(A)** Effects of overexpression of EcG3BP1 on IFN-3 and ISRE promoter activities. **(B)** and **(C)** Overexpression of G3BP1 increased the expression of interferon related cytokines and pro-inflammatory factors. pEGFP-EcG3BP1 was transfected into GS cells, which were collected at 36 h to detect the expression of interferon related cytokines (IRF3, ISG15, and ISG56) and pro-inflammatory factors (IL-1β, IL-8, and TNF-α) by qRT-PCR (*p < 0.05, **p < 0.01).

When EcG3BP1 was co-transfected into GS cells with ISRE-Luc and IFN-Luc reporter genes, EcG3BP1 significantly increased IFN-3 and ISRE promoter activity compared with the transfected empty (**Figure 7A**). To further elucidate the potential mechanism of EcG3BP1 in viral infection, the roles of EcG3BP1 in host interferon immunity and inflammatory response were evaluated by qRT-PCR. After pEGFP-EcG3BP1 was transfected into GS cells for 36 h, the expression levels of IFN and interferon related genes such as IRF3, ISG15, and ISG56 were significantly up-regulated compared with controls (**Figure 7B**). In addition, transcription levels of pro-inflammatory factors such as interleukin-1β (IL-1 β), IL-8, and tumor necrosis factor alpha (TNF-α) were also increased in cells overexpressing EcG3BP1 (**Figure 7C**).

## Discussion

When eukaryotes are stimulated by external pressures such as oxidative stress and virus infection, translation of some proteins is suspended in the cytoplasm, resulting in the formation of dense dynamic granular structures. Most viruses can use host cell resources to complete their own viral protein synthesis, which restricts host cell protein translation and causes SGs to form, which in turn helps cells survive by resisting viral infection (4). We found that when GS cells were infected with RGNNV for 6 h, SGs began to form. The number of SGs increased and persisted during RGNNV infection, indicating that RGNNV infection induced the formation of stable SGs. The formation of SGs caused by different types of viruses can follow four different patterns: infection induces stable SG formation, infection induces and then inhibits SGs formation, SG generation and depolymerization appear alternately, and inhibition of SGs formation. For example, SGs induced by respiratory syncytial virus can remain stable during infection. When cells were infected with inactivated RGNNV, the ability to induce SG formation was almost lost, suggesting that only RGNNV with normal replication function could induce SG formation.

SG markers include G3BP1, TIA-1, eIF3η, eIF4A, and other components, and eIF3η and eIF4A are classic SG markers (32). The type of SG induced by RGNNV can be identified based on the classical SG marker protein eIF3η. Our results showed that overexpression of EcG3BP1 and eIF3η resulted in punctiform aggregations of these proteins in virus-infected cells. The co-localization of the proteins in the aggregations indicated that RGNNV-induced SGs belonged to the classical type. Classic SGs can be depolymerized by CHX, which inhibits the formation of SGs. We found that overexpression of EcG3BP1 in GS cells induced SG formation and co-localization with the endogenous stress granule marker protein TIA-1 and the viral CP protein under all three test conditions. In CHX-treated cells, however, the number of observable SGs was significantly lower than that of the control group, further indicating that the SGs belonged to the classical type.

Viral infection of host cells leads to translation termination, phosphorylation of eIF2α, and formation of SGs. SGs have an antiviral effect, thus, most viruses do not induce the production of SGs to avoid their antiviral function (33). Some viruses, such as herpes simplex virus 1 (34), inhibit the phosphorylation of eIF2α, and some are even eIF2α-independent. Previous studies reported that the phosphorylation of eIF2α was up-regulated during RGNNV infection. In our study, we did not detect significant differences in the number of SGs in the HRI, GCN2, and PRK groups compared with the control group, whereas PERK-1 significantly inhibited the production of SGs. The western blot results confirmed this conclusion. Studies have shown that PRRSV infection of MARC-14-5 cells induces the endoplasmic reticulum stress response and phosphorylation of eIF2α by activation of PERK (35, 36). In summary, determining whether formation of SGs induced by RGNNV infection is related to endoplasmic reticulum stress needs further study.

G3BP1 is a prerequisite for the formation of SGs, as the dimerization of its RRM domain is important during the assembly process. The RRM domain contains two conserved sequences, RNP1 and RNP2, which can bind to RNA to form special structures. The interaction between RRM and RNA is affected by the binding of RRM to other proteins (37). Our results suggested that overexpression of G3BP1 alone can lead to the aggregation of SGs, irrespective of the stress treatment and with or without external stress. As in humans, the conserved domain NTF2 is also necessary for the formation of SGs (21). In chickens, however, overexpression of the NTF2 structural domain played a negative regulatory role. We found that SGs were not produced when the two conserved structural domains of G3BP1 were overexpressed in GS cells, indicating that the independent expression of RRM and NTF2-like domains affected the induction ability of G3BP1. Additionally, only full-length G3BP1 could induce SG formation. In our current study, the important roles of G3BP1 in SG formation were initially analyzed and the conditions under which G3BP1 mediates SG formation were also identified.

G3BP1 connects the regulation of gene expression inside cells with external cell signals by changing the transcript. For example, in some cancers, overexpression of G3BP1 can regulate Ras, p53 (38), and transforming growth factor β signaling pathways (39), promote tumor cell proliferation, and inhibit apoptosis. During flavivirus infection, G3BP1 intrinsically regulates IFITM2 (40), ISG15, and PKR (41), thereby affecting the expression of type I IFNs. In addition, G3BP1 can not only regulate viral transcription, but it also interacts with viral RNA. However, few studies have focused on the mechanism responsible for the antiviral role of G3BP1 in lower vertebrates. BLAST analysis showed that EcG3BP1 contains the NTF2 domain and an RRM domain. The NTF2-like domain mediated the dimerization of G3BP1 to produce SGs and was related to nuclear transport and localization. In other studies, the NTF2-like domain of G3BP1 was found to interact with the NSP3 proteins of herpes simplex virus and Sindbis virus (42, 43).

EcG3BP1 encodes 495 amino acids and shares 93.5% and 65.9% homology with *S. aurata* and *H. sapiens*, respectively. EcG3BP1 was expressed in various tissues of grouper, but there were differences in expression in different tissues, with the highest expression in the spleen. The subcellular localization analysis revealed cytoplasmic distribution of EcG3BP1 in GS cells, including dot-like aggregations. Overexpression of G3BP1 in chickens also produced dot-like aggregation, which was related to the formation of SGs and indicated that G3BP1 is closely associated with SGs. In RGNNV-infected cells, the expression of EcG3BP1 increased during viral infection and peaked at 30 h post-infection. Scholte et al. reported that G3BP1 promoted the infection of Chikungunya virus, while knockdown significantly affected viral replication; these results suggested that G3BP1 may be associated with innate immunity after virus infection (44).

EcG3BP1 was overexpressed into GS cells and infected with RGNNV, and the effect of EcG3BP1 on viral replication was examined at the transcriptional and translational levels, respectively. Overexpression of EcG3BP1 inhibited RGNNV infection-induced replication *in vitro*. Hepatitis C virus (HCV) NS_5B protein and RNA of HCV interact with G3BP1 to regulate HCV transcription in a certain way, which suggests that G3BP1 is involved in the composition of the HCV replicase complex (44–46). In those studies, researchers found that HCV replication was significantly inhibited after G3BP1 knockdown, suggesting that G3BP1 can promote HCV proliferation. In contrast, studies of mammalian orthorectic virus and porcine epidemic diarrhoea virus (PEDV) showed that interfering with G3BP1 enhanced PEDV replication in Vero cells and that overexpression of G3BP1 reduced PEDV replication (47, 48). These results suggest that fish G3BP1 and mammal G3BP1 may have similar roles in virus replication.

To further elucidate the potential roles of EcG3BP1 in viral infection, host IFN immunity and the inflammatory response were evaluated in EcG3BP1 overexpressed cells infected with RGNNV. Expression of IFN-stimulating genes such as IRF3, ISG15, and ISG56 as well as pro-inflammatory cytokines including interleukin-1 β, IL-8, and TNF-α was up-regulated. The results indicated that EcG3BP1 inhibits RGNNV infection through positive regulation of IFN immunity and the pro-inflammatory response. Further analysis of the reporter genes showed that EcG3BP1 up-regulated IFN-3 and ISRE promoter activities. However, the regulatory mechanisms responsible for the IFN and inflammatory response related to EcG3BP1 in viral infection require further exploration.

In conclusion, RGNNV infection induced grouper spleen (GS) cells to produce SGs. The SGs particles co-located with the classic SG marker protein eIF3η. In addition, a SG marker protein RAS GTPase-activating protein (SH3 domain)-binding protein 1 (G3BP1) from orange-spotted grouper (*E. coioides*) (EcG3BP1) was cloned, and the roles of EcG3BP1 in innate immunity were investigated. The results showed that EcG3BP1 plays crucial roles in virus replication by up-regulating the interferon and inflammatory response.

## Data Availability Statement

The author selected the following statement: The datasets presented in this study can be found in online repositories. The names of the repository/repositories and accession number(s) can be found in the article/supplementary material.

## Author Contributions

Conceptualization, JW and QQ. Methodology, MS. Software, MS. Validation, JL, SK and LZ. Investigation, ZX, JL, SW, HC, LX, XZ. Writing—original draft preparation, MS. Writing—review and editing, JW. Supervision, QQ. Funding acquisition, QQ. All authors have read and agreed to the published version of the manuscript

## Funding

This research was funded by the National Key R&D Program of China (2018YFD0900500 and 2018YFC0311300), China Agriculture Research System of MOF and MARA(CARS-47-G16), the Key-Area Research and Development Program of Guangdong Province (2021B0202040002), Laboratory of Lingnan Modern Agriculture Project (NT2021008), and Innovation Group Project of Southern Marine Science and Engineering Guangdong Laboratory (Zhuhai) (311021006).

## Conflict of Interest

The authors declare that the research was conducted in the absence of any commercial or financial relationships that could be construed as a potential conflict of interest.

## Publisher’s Note

All claims expressed in this article are solely those of the authors and do not necessarily represent those of their affiliated organizations, or those of the publisher, the editors and the reviewers. Any product that may be evaluated in this article, or claim that may be made by its manufacturer, is not guaranteed or endorsed by the publisher.
